# Molecular Poltergeists: Mitochondrial DNA Copies (*numts*) in Sequenced Nuclear Genomes

**DOI:** 10.1371/journal.pgen.1000834

**Published:** 2010-02-12

**Authors:** Einat Hazkani-Covo, Raymond M. Zeller, William Martin

**Affiliations:** 1 National Evolutionary Synthesis Center, Durham, North Carolina, United States of America; 2 Mathematics Undergraduate Program, Duke University, Durham, North Carolina, United States of America; 3 Institut für Botanik III, Heinrich-Heine Universität Düsseldorf, Düsseldorf, Germany; Fred Hutchinson Cancer Research Center, United States of America

## Abstract

The natural transfer of DNA from mitochondria to the nucleus generates nuclear copies of mitochondrial DNA (*numts*) and is an ongoing evolutionary process, as genome sequences attest. In humans, five different *numts* cause genetic disease and a dozen human loci are polymorphic for the presence of *numts*, underscoring the rapid rate at which mitochondrial sequences reach the nucleus over evolutionary time. In the laboratory and in nature, *numt*s enter the nuclear DNA via non-homolgous end joining (NHEJ) at double-strand breaks (DSBs). The frequency of *numt* insertions among 85 sequenced eukaryotic genomes reveal that *numt* content is strongly correlated with genome size, suggesting that the *numt* insertion rate might be limited by DSB frequency. Polymorphic *numts* in humans link maternally inherited mitochondrial genotypes to nuclear DNA haplotypes during the past, offering new opportunities to associate nuclear markers with mitochondrial markers back in time.

## Introduction

Endosymbiosis is germane to eukaryote evolution, and gene transfers from organelles to the nucleus were an important mechanism of genetic variation that helped to forge the prokaryote-to-eukaryote transition [1]–[3]. Though DNA can be experimentally relocated from organelles to the nucleus in the laboratory [4],[5], the more far-reaching experiment is the one ongoing in nature over evolutionary time. All genome sequences from eukaryotes that have DNA in their mitochondria (for exceptions see [6]) harbour evidence for the ongoing process of organelle-to-nuclear DNA transfer in the form of nuclear copies of mitochondrial and, in the case of plants, chloroplast DNA [7]. Genome sequences from those eukaryotes that have lost their mitochondrial DNA altogether still harbour evidence for gene transfers from the mitochondrion during the early phases of eukaryote history [3],[6],[8].

The story of gene wanderings, from organelles to the nucleus during recent evolutionary time, started with the report of a gene sequence that was present in both the nuclear and the mitochondrial genome in *Neurospora* [6],[9]. That set the stage for a deluge of other examples for Òpromiscuous DNAÓ [10]. The term *numts* (pronounced “new-mights”), for nuclear sequence of mitochondrial origin, was coined [11] to designate such DNA, which was often discovered inadvertently in the search for bona fide mtDNA (Box 1). Since that time, *numt* population polymorphism [12],[13] and *numt* variation among human siblings has been found [14]. In the case of photosynthetic species, the corresponding sequences are called *nupts* (nuclear copies of plastid DNA, pronounced “new-peats”). With the recent eruption of eukaryotic genome data, it is opportune to take a look at the prevalence and properties of *numts* in sequenced eukaryotic genomes.

**Box 1. *Numts* Cause Confusion**

Due to their sequence similarity to mitochondrial DNA, *numts* are responsible for many instances of misidentification, both in mitochondrial disease studies and phylogenetic reconstruction.

**Mitochondrial Disease Confusions**

*Numts* are common in humans. As a result, *numt* variation is continuously mis-reported as mitochondrial mutations in patients [82],[83]. At least one *numt* (5,842 bp *numt* on chromosome 1) was erroneously implicated in causing diseases, such as low sperm motility [84] and cystic fibrosis (see details in [82]). Even the HapMap data first classified this *numt* as mitochondrial variation [85]. If you have this variant in your genome, there is no cause for concern because it is not mitochondrial variation, it is a nuclear pseudogene.

**DNA Barcoding and Phylogenetic Confusion**

Mitochondrial DNA is commonly used as a marker for molecular systematics, phylogeny and for species diagnosis (“DNA barcoding”). The DNA barcoding technique for animals aims to identify organisms by using a short fragment of mitochondrial cytochrome c oxidase I (COI) gene [86],[87]. *Numts* are a major challenge in using mitochondria for these purposes [88],[89]. It was suggested that because of *numt*s, the barcoding approach is unreliable, at least in primates [90]. Recently, DNA barcoding among arthropods was found to overestimate the number of species when *numts* are coamplified [91], showing that *numts* introduce serious ambiguity into the DNA barcoding paradigm as arthropods are one the major phyla studied in taxonomy.

**Ancient DNA That Isn't Ancient**

The report that 80-million-year-old dinosaur bones harboured DNA [92] made quite a splash in its time, appearing a year after the filming of Jurassic Park. But it did not take long to uncover the real source of dinosaur bone DNA; it was a mtDNA pseuodgene in the human nuclear genome [93],[94], now called a *numt*. Newer findings even implicate *numt*s in reports of horizontal gene transfer among plants [95].

## The Human Genome—Visible, Ongoing *Numt* Transfer

Sequenced eukaryotic genomes can be readily scanned for *numts* using standard data-mining tools. Attempts to identify *numts* solely with computer methods started with partial genome sequences of plants and yeast [15],[16] followed by scanning of the full genomes of human, fruitfly, *Plasmodium*, and *Caenorhabditis* [17],[18]. Various studies focused on the identification of *numts* specifically in the human genome [18]–[20]. The number of human *numts* was reported with values ranging from 286 to 612 depending on the search parameters and depending on how closely related were combined hits into a single *numt* contig. Later calculations based on *numts* from both human and chimpanzee suggested an intermediate number of 452 *numts* [21]. Some of the human *numts* stem from independent insertion events from the mitochondrion, whereas others are the results of tandem duplications [19] or subsequent segmental duplications. Older *numts* appear in more copies than recent ones [22].

**Figure 1:**
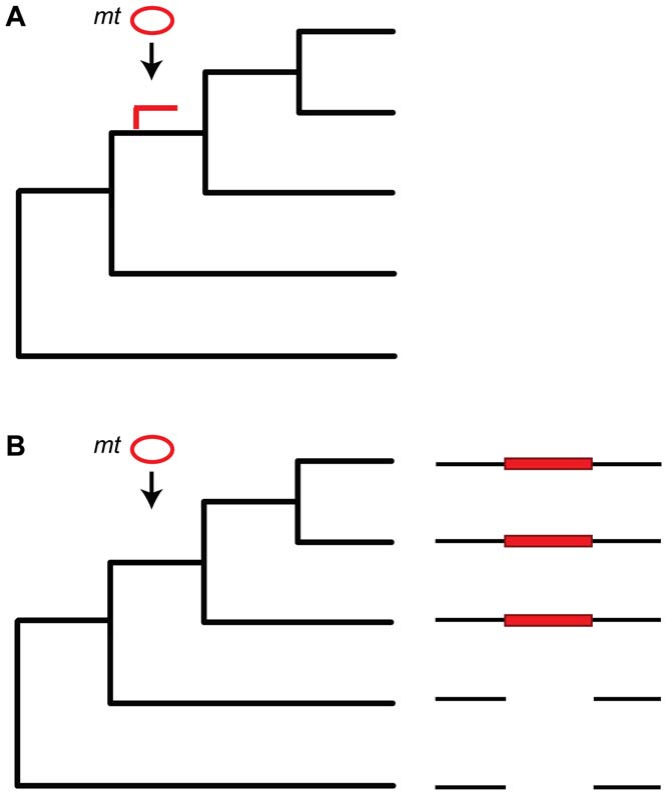
Dating *numt* insertion. (A) Dating *numt* insertion based on a mitochondrial phylogenetic tree (black branches). An arrow indicates time of insertion and the *numt* branch is shown in red. The methodology can be used only in species where the mitochondrial rate of evolution is lower than the nuclear rate of evolution (e.g., mammals but not plants) and when the *numts* are long enough (>1 kb) to carry enough evolutionary signal. (B) Dating *numt* insertion based on patterns of presence and absence on a phylogeny. Few nuclear genomes and their genome alignment are used to identify *numt* insertions. Species that share the descendant from the common ancestor where the transfer occurred include the *numts* (red rectangle) whereas this *numt* is missing in the others.

The largest human *numt* covers 90% (14,654 bp) of the human mitochondrial genome [18]. Comparisons involving primate mitochondrial sequences allow one to approximately date the timing of insertion for long *numts* [22],[23] (Figure 1A). Such dating is based on the observation that the mean evolutionary rate in primate mitochondrial genomes is about ten times higher than that in the nuclear genome [24]–[26]. Therefore *numts* inserted into the nucleus decelerate their evolutionary rate and become “molecular fossils” resembling ancestral mitochondrial fragments [27],[28]. With the possible exception of an event involving either rapid post-insertion duplication [22] or rapid insertion per se [23] during the time corresponding to the Platyrrhini–Catarrhini divergence, *numt* insertion appears to have been more or less continuous over time in the lineages leading to the human genome [18],[22],[23].

Phylogenetic and PCR amplification studies in humans suggest that the rate of *numt* insertion is ∼5.1–5.6×10^−6^ per germ cell per generation, or that every two human haploid genomes should be polymorphic for at least two *numt* loci [23],[29],[30]. Ricchetti et al. [30] used a PCR analysis with primers from both the nuclear flanking regions and the *numt* sequence to identify recent *numt* insertions that appear only in the human genome but not in the chimpanzee genome. Based on whole genome alignments, more than 80% of the *numts* in the human and chimpanzee genomes were found to be orthologous in that they are present at the same loci in the two species [21], but non-orthologous *numts* stemming from recent *numt* insertions, deletions, and tandem duplications were also identified. Current estimates have it that there are 40 and 68 species-specific insertions in the human and chimpanzee lineages, respectively [31].

**Figure 2:**
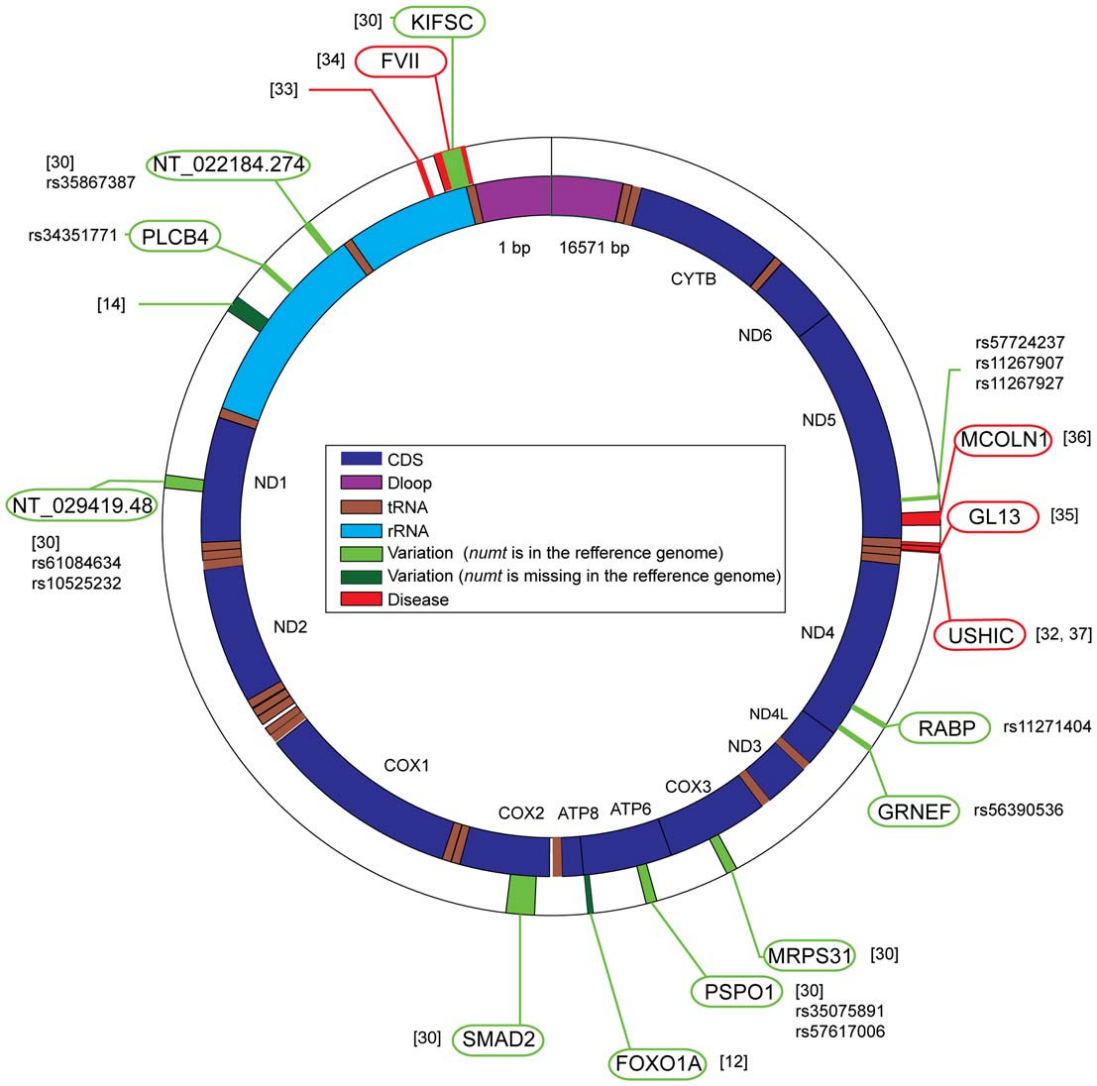
Human polymorphic *numts* and *numts* that cause diseases. Human mitochondrial DNA (NC_001807) is shown in the inner circle, and *numt* insertions are shown in the outer circle. Polymorphic *numts* are shown in light green (*numts* exist in the reference genome) or dark green (*numts* are missing from the reference genome). *Numts* causing disease are shown in red. In each case, the reference and the SNP accession numbers (if available) are given. When a *numt* is inserted within gene, the gene name is indicated (green and red ellipses for polymorphic *numts* and for *numts* causing disease, respectively).

Eight loci that are polymorphic for *numts* have been reported in humans so far [12],[14],[30] using PCR-based approaches. We have uncovered four additional polymorphic *numts* by searching the human dbSNP database for *numts* that appear in the reference human genome and are missing in the variation data. Overall, about a third of human-specific *numts* (12/40) are variable (Figure 2). Ten out of the 12 polymorphic *numts* appear in genes or in predicted genes [30]. With the increasing availability of structural variation data in populations, the number of loci polymorphic for *numts* is predicted to increase, and it should be possible to identify variable more *numts* that are missing in the reference genome(s) but appear in the variation data.

## *Numts* and Diseases

Integration of *numt*s not only appears as neutral polymorphism but, more rarely, is also associated with human diseases [32]; five cases are currently known (Figure 2). One involved a 41-bp mtDNA insertion at the breakpoint junction of a reciprocal translocation between chromosome 9 and 11 [33], the remaining cases involve insertion of mtDNA into genes. A splice site mutation in the human gene for plasma factor VII that causes severe plasma factor VII deficiency (bleeding disease) results from a 251-bp *numt* insertion [34]. A rare case of Pallister-Hall syndrome in which a 72-bp *numt* insertion into exon 14 of the GLI3 gene causes a premature stop codon, is associated with Chernobyl [35]. A case of mucolipidosis IV in which a 93-bp segment was inserted into exon 2 of *MCOLN1*, eliminated proper splicing of the gene [36]. As the last known example, a 36-bp insertion in exon 9 of the *USH1C* gene associated with Usher syndrome type IC [37] is a *numt* [32]. As in other cases of *numt* insertions, the mitochondrial genome remains intact in the afflicted individuals.

## More Genomes, More *Numts*

Beyond humans, the whole genome repertoire of *numts* has been estimated in various species including yeasts [38], rodents [39], plants [40], and honeybees [41],[42]. *Numts* show not only different frequencies in different genomes, but also different size distributions [43],[44]. *Numts* are abundant in plants, where the longest *numt* known so far, a 620-kb partially duplicated insertion of the 367-kb mtDNA of *Arabidopsis thaliana*, was reported [45].

The honeybee genome is currently the record-holder for *numt* frequency among metazoans so far [41],, although their *numts* are relatively short. Since the last genome-wide survey encompassing 13 nuclear genomes [44], 72 new eukaryotic genome sequences have become available for study. Table 1 summarizes the *numt* repertoire in 85 fully sequenced genomes including 20 fungi, 11 protists, 7 plants, and 47 animals, for which both nuclear and mitochondrial genomes are available, reporting the number of BLAST nucleotides that were found in the genome (BLASTN of entire mitochondria against the genome using e-score of 0.0001). Some mitochondrial genomes (those of plants, for example), contain repetitive sequences, such that a single nuclear fragment can be found by BLAST to match multiple mitochondria pieces, a source of differences between tabulations in earlier reports. Each nuclear nucleotide appearing in Table 1 is unique and is counted only once even if the corresponding *numt* matches multiple mtDNA regions.

**Table 1:** Blast analysis of 85 mitochondria against their nuclear genomes (BlastN, e-score = 0.0001).

	*numt* content (Kb)	No. of BLAST hits	mtDNA Accession	mt Length (Kb)	Number of mt proteins	*numt* content in nuclear genome (%)	Other estimates (Kb)
**Animals**							
*Aedes aegypti*	67.974	418	NC_010241	16.655	13	0.0085	
*Anopheles gambiae*	0	0	NC_002084	15.363	13	0.0000	0 [44]
*Apis mellifera*	172.131	1790	L06178	16.343	13	0.0861	237, 272 [41],[42]
*Bombyx mori*	8.304	53	NC_002355	15.643	13	0.0016	
*Bos taurus*	69.864	279	NC_006853	16.338	13	0.0023	
*Branchiostoma floridae*	0	0	NC_000834	15.083	13	0.0000	
*Brugia malayi*	22.387	180	NC_004298	13.657	12	0.0204	
*Caenorhabditis briggsae*	14.39	73	NC_009885	14.42	12	0.0138	
*Caenorhabditis elegans*	0.126	1	NC_001328	13.794	12	0.0001	0.1 [44]
*Canis lupus familiaris*	63.513	281	NC_002008	16.727	13	0.0026	
*Cavia porcellus*	479.657	596	NC_000884	16.801	13	0.0141	
*Ciona intestinalis*	11.771	64	NC_004447	14.79	13	0.0076	11 [44]
*Ciona savignyi*	0	0	NC_004570	14.737	12	0.0000	
*Danio rerio*	0	0	NC_002333	16.596	13	0.0000	
*Daphnia pulex*	8.298	91	NC_000844	15.333	13	0.0037	
*Dasypus novemcinctus*	72.24	89	NC_001821	17.056	13	0.0024	
*Drosophila melanogaster*	10.331	50	NC_001709	19.517	13	0.0057	0.5 [44]
*Drosophila sechellia*	22.507	96	NC_005780	14.95	13	0.0150	
*Drosophila simulans*	2.747	15	NC_005781	14.972	13	0.0020	
*Drosophila yakuba*	10.066	47	NC_001322	16.019	13	0.0056	
*Echinops telfairi*	388.74	325	NC_002631	16.549	13	0.0130	
*Equus caballus*	54.72	203	NC_001640	16.66	13	0.0018	
*Erinaceus europaeus*	413.569	334	NC_002080	17.447	13	0.0138	
*Felis catus*	147.361	232	NC_001700	17.009	13	0.0049	298 [47]
*Gallus gallus*	1.52	12	NC_001323	16.775	13	0.0001	
*Gasterosteus aculeatus*	17.347	5	NC_003174	15.742	13	0.0026	
*Homo sapiens*	263.478	871	NC_001807	16.571	13	0.0087	
*Loxodonta africana*	127.551	149	NC_000934	16.866	13	0.0043	
*Macaca mulatta*	261.622	804	NC_005943	16.564	13	0.0087	
*Monodelphis domestica*	2093.63	1859	NC_006299	17.079	13	0.0698	
*Mus musculus*	37.67	137	NC_005089	16.299	13	0.0015	53 [44]
*Ochotona princeps*	98.16	162	NC_005358	16.481	13	0.0033	
*Ornithorhynchus anatinus*	244.198	271	NC_000891	17.019	13	0.0081	
*Oryctolagus cuniculus*	183.38	182	NC_001913	17.245	13	0.0052	
*Oryzias latipes*	17.143	16	NC_004387	16.714	13	0.0021	
*Pan troglodytes*	294.682	1065	NC_001643	16.554	13	0.0095	
*Petromyzon marinus*	27.2	85	NC_001626	16.201	13	0.0013	
*Pongo abelii*	218.739	954	NC_002083	16.499	13	0.0073	
*Rattus norvegicus*	6.023	49	NC_001665	16.313	13	0.0002	6 [44]
*Schistosoma mansoni*	28.747	53	NC_002545	14.415	12	0.0106	
*Strongylocentrotus purpuratus*	2.041	10	NC_001453	15.65	13	0.0003	
*Takifugu rubripes*	16.032	7	NC_004299	16.447	13	0.0041	5.6 [44]
*Tetraodon nigroviridis*	10.783	8	NC_007176	16.462	13	0.0031	
*Tribolium castaneum*	31.174	139	NC_003081	15.881	13	0.0156	
*Trichoplax adhaerens*	0.095	1	NC_008151	43.079	17	0.0002	
*Tupaia belangeri*	463.794	461	NC_002521	16.754	13	0.0155	
*Xenopus tropicalis*	10.985	17	NC_006839	17.61	13	0.0006	
**Plants**							
*Arabidopsis thaliana*	305.602	820	NC_001284	366.924	117	0.2564	198 [44]
*Chlamydomonas reinhardtii*	2.85	45	NC_001638	15.758	8	0.0029	
*Oryza sativa Indica Group*	823.923	5357	NC_007886	491.515	54	0.1768	
*Ostreococcus tauri*	0.708	7	NC_008290	44.237	43	0.0057	
*Physcomitrella patens*	76.339	340	NC_007945	105.34	42	0.0149	
*Sorghum bicolor*	539.091	2716	NC_008360	468.628	32	0.0709	
*Zea mays subsp mays*	71.074	376	NC_007982	569.63	165	0.0030	
**Fungi**							
*Aspergillus niger*	0.298	6	NC_007445	31.103	16	0.0008	
*Aspergillus oryzae*	25.47	64	NC_008282	29.202	NA	0.0688	
*Aspergillus terreus NIH2624*	0.89	11	NT_165950	32.827	NA	0.0025	
*Candida albicans SC5314*	0.078	214	NC_002653	40.42	13	0.0005	
*Candida glabrata CBS 138*	0.656	5	NC_004691	20.063	11	0.0053	
*Cryptococcus neoformans var grubii*	46.296	66	NC_004336	24.874	12	0.2315	
*Debaryomyces hansenii*	7.872	119	NC_010166	29.462	18	0.0644	9 [38]
*Gibberella zeae*	0.544	3	NC_009493	95.676	50	0.0014	
*Kluyveromyces lactis*	0.319	6	NC_006077	40.291	9	0.0030	0.4 [38]
*Mycosphaerella graminicola*	0.101	2	NC_010222	43.964	22	0.0003	
*Paracoccidioides brasiliensis*	0.526	8	NC_007935	71.335	17	0.0018	
*Phaeosphaeria nodorum SN15*	77.142	108	NC_009746	49.761	19	0.2079	
*Podospora anserina*	0.455	3	NC_001329	94.192	53	0.0013	
*Rhizopus oryzae*	0.172	2	NC_006836	54.178	24	0.0004	
*Saccharomyces cerevisiae*	0.983	18	NC_001224	85.779	19	0.0081	1.2, 2.3 [38],[44]
*Schizosaccharomyces japonicus*	0.093	1	NC_004332	80.059	10	0.0007	
*Schizosaccharomyces octosporus*	1.245	13	NC_004312	44.227	14	0.0089	
*Schizosaccharomyces pombe*	1.494	16	NC_001326	19.431	10	0.0120	1.6 [38]
*Ustilago maydis*	58.575	92	NC_008368	56.814	26	0.2857	
*Yarrowia lipolytica*	0.908	21	NC_002659	47.916	24	0.0044	2 [38]
**Protists**							
*Cyanidioschyzon merolae*	0	0	NC_000887	32.211	34	0.0000	
*Dictyostelium discoideum*	0.175	2	NC_000895	55.564	42	0.0005	
*Emiliania huxleyi*	0.178	1	NC_005332	29.013	21	0.0001	
*Monosiga brevicollis*	0	0	NC_004309	76.568	32	0.0000	
*Naegleria gruberi*	0	0	NC_002573	49.843	46	0.0000	
*Phytophthora infestans*	111.22	675	NC_002387	37.957	40	0.0463	
*Phytophthora ramorum*	0.757	8	NC_009384	39.314	43	0.0012	
*Phytophthora sojae*	9.948	152	NC_009385	42.977	47	0.0105	
*Plasmodium falciparum*	0.144	5	NC_002375	5.967	3	0.0006	
*Tetrahymena thermophila*	1.457	36	NC_003029	47.577	45	0.0007	
*Thalassiosira pseudonana*	0	0	NC_007405	43.827	35	0.0000	

For each organism the number of BLAST hits as well as the unique number of bases in genomes is given (i.e. a base in the genome that has a BLAST hit to two repetitive mitochondria pieces it is count only once in *numt* content). Other available *numt* estimates are indicated with their references, where the corresponding search parameters are given.

*Numts* are common in all groups that were examined. The *numt* content of these genomes varies from no detectable *numts* in eight species to more than 500 kb in three genomes. As noted by Richly and Leister [44] the fraction of the nuclear genome represented by *numts* is usually less than 0.1%, with the higher proportions of *numts* appearing in plants and yeast [15],, two groups that each include a few genomes consisting to >0.1% out of *numt*s. At first sight, 0.1% might not seem like much, but *numt* sequences are constantly becoming undetectable through mutation and deletion, such that 0.1% represents a steady state level of recently incorporated and detectable *numt*s at any given point in time.

For organisms that have only one mitochondrion, such as *Cyanidioschyzon*, the absence of *numts* makes sense, because if an organelle must lyse in order for DNA to escape to the nucleus, then more than one organelle per cell (one for gene transfer and one for healthy progeny) would be required for the DNA to escape [46]. The absence of *numts* in the present releases of several animal genomes, from insects to vertebrates, is an exception in that regard, but annotations can change over time. The highest total *numt* content was found in the opossum *Monodelphis domestica*, whose genome sequence contains over 2000 kb of *numt* nucleotides. However, most opossum *numts* do not map to known chromosome arms, and some fraction of these may turn out to be true mitochondrial sequences. In plants, the highest *numt* content appears in *Oryza sativa Indica* group with more than 800 kb of *numts*. Among fungi, the highest *numt* content appears in *Phaeosphaeria nodorum* with 77 kb, and in protists the highest *numt* content so far appears in *Phytophthora infestans* with 111 kb.

The number of *numts* one detects can change with search strategy, genome version and level of genome completion. For example, when calculated in 2009, the genome of *Arabidopsis* has 54% more total *numt* length (305.6 kb) than it did five years ago (198 kb) [44], in part because some *numts* were initially removed during the annotation process [46]. Similarly, the *numt* content in the *Drosophila melanogaster* genome has grown from 0.5 kb in 2004 to a current value of 10.3 kb (Table 1), corresponding to a roughly 20-fold increase. These differences are due to changes in the curation of the available genome sequence data. For example, the current version of the *D. melanogaster* genome includes 4.7 Mb of heterochromatic sequence that was previously unavailable. By contrast, in the cat genome, not all of the *numts* reported by Lopez et al. (1994) [11] are identified using the standard parameters, and a careful analysis of *numts* [47] suggests that the genome might include as much as double the number of *numts* identified here. Other available assessments of *numt* content in genomes are shown in Table 1.

**Figure 3:**
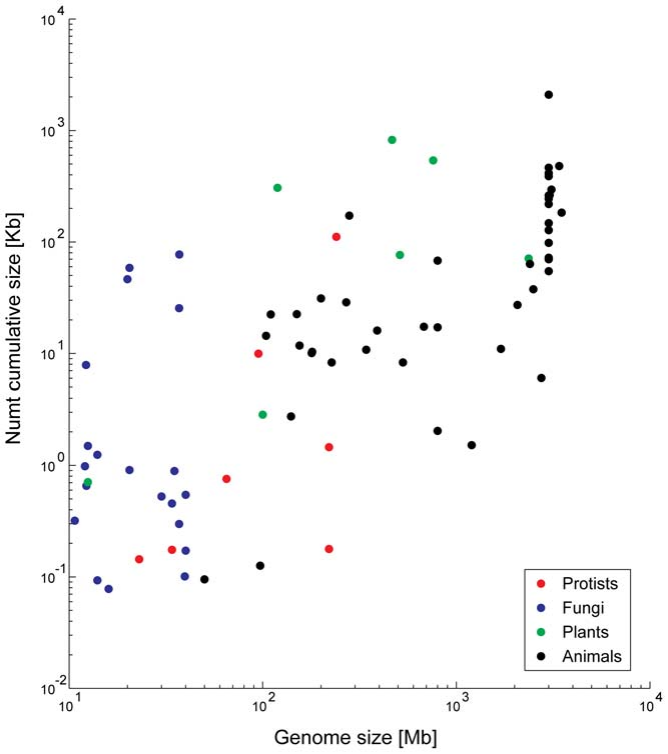
*Numt* content is correlated to genome size. A log–log scale graph showing the dependency between *numt* content in genomes and genome size. Information regarding genome size is from http://www.ncbi.nlm.nih.gov/genomes/leuks.cgi.

The data from 85 genomes reveal a strong correlation between genome size and total *numt* content (Spearman non-parametric rho = 0.67, P = 2.77×10^−12^). Bensasson et al. [17],[43] suggested that such a correlation might exist for metazoans because genomes with more non-coding DNA will have more *numts* (see below). Early searches detected no such correlations [44], probably owing to the small sample size. A fresh look at the data reveals the predicted correlation, which however seems to explain mainly the differences between small and big genomes (Figure 3), as it disappears when considering only genomes smaller than 200 Mb. No correlations appear between *numt* content and mitochondrial genome size, even when *numt* content is normalized by the nuclear genome size. Three different processes can thus contribute to the differences in *numts* between species—the frequency of mitochondrial transfer, the amount of chromosomal integration, and the dynamics of post-insertion processes, such as duplications and deletions affecting all DNA as part of bulk genome evolution.

## Mechanism of *Numt* Insertions

**Figure 4:**
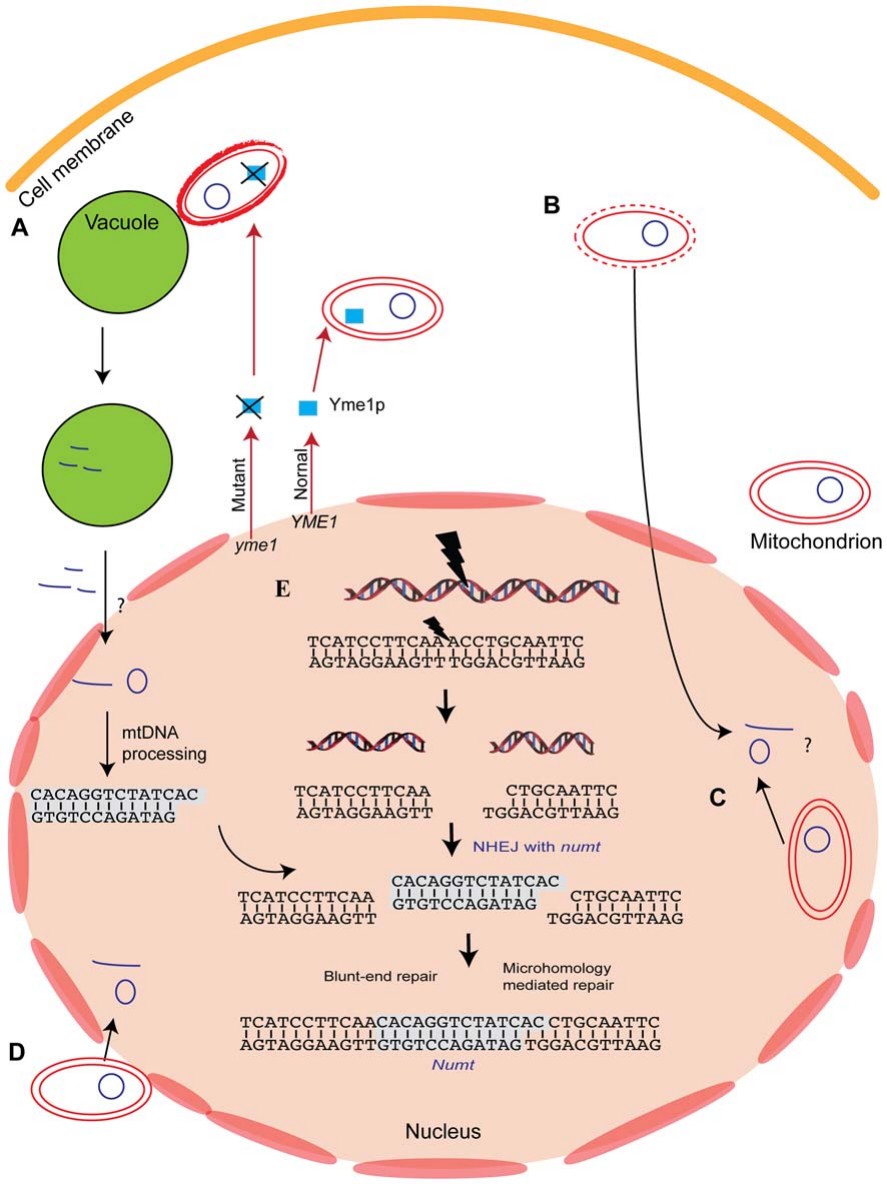
Mechanism of *numt* insertion. Mitochondrial DNA has been suggested to get into the nucleus via a few different pathways. (A) The most supported pathway so far involve the degradation of abnormal mitochondria [53]. Several *yme* (yeast mitochondrial escape) strains show high level of DNA escape to the nucleus. *yme1* mutant cause the inactivation of YMe1p protein, a mitochondrial-localized ATP-dependent metallo-protease leading to high escape rate of mtDNA to the nucleus. Mitochondria of *yme1* strain are taken up for degradation by the vacuole more frequently than the wild-type strain. Other pathways to get mitochondrial DNA into the nucleus were suggested including: (B) lysis of mitochondrial compartment, (C) encapsulation of mitochondrial DNA inside the nucleus, (D) direct physical association between the mitochondria and the nucleus and membrane fusions. (E) Mitochondrial DNA that enters the nucleus can integrate into nuclear chromosomes. mtDNA integrated into the chromosome during the repair of DSBs in a mechanism known as non-homologous end-joining (NHEJ). The insertion involves two DSB repair events. Each can be repaired with or without the involvement of short microhomology. In microhomology-mediated NHEJ, base-pair complements are available between the *numt* and the chromosome ends, similar to the sticky ends created by restriction enzymes.

For *numts* to persist in nuclear genomes, mitochondrial DNA must first physically reach the nucleus, then it must integrate into the nuclear chromosome, with intragenomic dynamics of amplification, mutation, or deletion following. Work so far has focused on the escape of DNA from the mitochondria and on the integration of mtDNA within the nucleus but not on its physical entrance into the nucleus (the notion that nuclear chromosomes should actively pluck mtDNA from the organelle seems unlikely enough to exclude). The current picture is summarized in Figure 4, but we are still far from understanding the full details.

### Export from the Mitochondria

Thorsness and Fox [48] utilized an assay to measure the rate of mtDNA escape to the nucleus in *S. cerevisiae*. Their assay was based on engineering the *URA3* gene, which is involved in uracil biosynthesis, from the nuclear genome to a plasmid that is maintained in the mitochondrion. During the propagation of such yeast strains carrying a nuclear *ura3* mutation, plasmid DNA that escapes from the mitochondrion to the nucleus complements the uracil biosynthetic defect, restoring growth in the absence of uracil, an easily scored phenotype. The rate of DNA transfer from the mitochondria to the nucleus was estimated as 2×10^−5^ per cell per generation [48]. Since the *URA3* gene carrying its own promoter was located on a plasmid, that experimental system only measured relocation of mtDNA into the nucleus and did not measure integration of the plasmid or mtDNA into the chromosome. In addition, it only measured the transport of the entire *URA3* gene, while shorter or other mitochondrial fragments went undetected. In a different experimental setup, mtDNA fragments joined to linear DNAs to form circular DNA plasmids. The integration frequency was suggested to be as high as 10^−3^ to 10^−4^, or that 1 in every 1,000–10,000 yeast cells might contain a new mitochondrial insertion [49]. The escape event was found to be intracellular, that is, lysis of cells in culture with mtDNA uptake by neighboring cells is not involved [50].

Increased rates of yeast mtDNA escape are observed in different conditions, including in cells that have been frozen and thawed, in cells that were grown in non-optimal temperature, and, when environment favors fermentation, as primary energy source. In addition, mutations in at least 12 nuclear loci called the *yme* (yeast mitochondrial escape) mutations, lead to an elevated rate of mtDNA escape to the nucleus [51],[52]. Some of the *yme* mutants have protein products that are mitochondrion-associated, and it has been suggested that perturbation in mitochondrial functions due to the alteration of gene products affect mitochondrial integrity, leading to mtDNA escape. In the case of the *yme1* strain, abnormal mitochondria are targeted for degradation by the vacuole, and this degradation increases mtDNA escape to the nucleus [53] in a process known as mitophagy [54],[55]. Cytological investigations have suggested several other pathways in diverse species (reviewed in [50]) including a lysis of the mitochondrial compartment, direct physical association between mitochondrial, and nuclear membranes [56], membrane fusions, and encapsulation of mitochondrial compartments inside the nucleus [57]. It was also suggested that the frequency of mitochondrial DNA transfer into the cytoplasm might change with the number of mitochondria within the germ-line [58], although experimental tests of this idea are so far lacking.

### Integration into the Nuclear Chromosome

The appearance of large mitochondrial segments within nuclear genomes including large fragments of non-coding regions [18],[20],[59] and no preference for transcribed over non-transcribed regions indicate that bulk organelle DNA, not transcripts or cDNAs, is integrated into nuclear chromosomes [60]. This is consistent with the observations from genetically engineered organelle-to-nucleus gene transfer experiments [4].

Based on *numt* integration sites, Blanchard and Schmidt [16] proposed that *numt*s are inserted into double-strand breaks (DSBs) by the non-homologous end joining (NHEJ) machinery. This was later borne out in an important study on yeast under conditions where homologous recombination was not possible [5]. Later analyses were consistent with the involvement of NHEJ in *numt* integration [30] in humans.

At the mechanistic level, there is a junction with chromosomal DNA to one side and mitochondrial DNA on the other at each end of a *numt*, and these junctions reflect the repair events at each end of the original chromosomal break (Figure 4). *Numts* can be integrated to chromosome ends with short microhomology of 1–7 bp, a NHEJ sub-mechanism known as microhomology-mediated repair. Insertion of *numt* can also occur without microhomology—a process known as blunt-end repair. It is possible to follow the details of *numt* insertion through NHEJ by analyzing the integration sites of recent *numt* insertions in primates. Comprehensive analysis of 90 recent *numt* insertions in human and chimpanzee suggest that 35% of the fusion points involve microhomology of at least 2 bp, thus, it appears that repair involving microhomology plays some role in *numt* integration but is not totally required [61].

Throughout the evolutionary history of human and chimpanzee, more than half of the DSBR events that involve *numts* do not show deletions. When deletions appear, they are very small [61]. This is surprising as the NHEJ mechanism underlying DSBR is inherently mutagenic; NHEJ repair events of similar break configurations without filler DNA (extrachromosomal DNA, i.e., *numts*) always involve small deletions and even in NHEJ reaction with filler DNA the frequency of deletions is significantly bigger (e.g., [62],[63] and referenced in [61]). This difference indicates that *numts* provide the end-joining machinery with a tool to seal breaks without the necessity to process the nuclear DNA further using a nuclease. Providing the repair system with *numts* as an alternative to nuclease activity might be important in cases where the structure of the DSB is chemically complex. Repairing complex DSBs without *numts* may require significant nuclease processing of chromosomal DNA, yielding a long stretch of single-strand DNA, which would potentially put the genome at risk for big deletions or translocations. It is thus possible that sealing DSBs with *numts* might abolish the risk of more deleterious DSBR [61]. There is a price tag for *numt*-mediated DSBR, though—an insertion. But this is a small price to pay for healing complex DSBs in non-coding regions. *Numts* are usually short; therefore their insertion might be less deleterious than the effects of exposed single-strand DNA. While the amount of *numts* in the genomes is too small to suggest that *numts* are significant in maintaining genome integrity by themselves, no other class of DNA fragments has yet been found that is captured into DSBs in a similarly healing role.

Despite its utility for mending DSBs in a manner that avoids deletions, mitochondrial DNA is not maintained during evolution as a spare parts warehouse for nuclear chromosomes. Instead it is, like chloroplast DNA, maintained because the membrane-associated electron transport functions of bioenergetic organelles demand that organelles have the capacity to immediately respond to redox imbalance at the level of individual organelles [64],[65]. Yet, when we consider the early phases of mitochondrial origins, the flux of DNA from the endosymbiont is generally thought to have had two major consequences for the evolution of eukaryotic chromosomes: it was a rich source of genetic novelties, on the one hand (for example eubacterial operational genes [66]), and a source of constructively disruptive forces on the other (for example introns [67]). As a third consequence, pieces of endosymbiont DNA might have been involved in DSB repair of the archaebacterial chromosomes of the host [68] right from the beginning as well.

## Post-Insertion Processes within the Nuclear Genome

*Numts* sometimes show a more complex pattern than a single mitochondria piece, and can include non-continuous pieces of mitochondrial DNA that can appear in different orientations [5],[19],[20]. In plants, such complex patterns of *numts* are very common and can involve shared clusters with *nupts* [29],[40]. It has been suggested that these complex patterns are the result of concatenation prior to insertion rather than the result of multiple *numt* or *nupt* insertions at insertional hotspots [69]. If they are, contrary to expectation, insertional (or DSBR) hotspots after all, they should turn out to be more polymorphic than other sites for *numt*s and/or *nupt*s in “1,000 genome”–type surveys; this will be something to look for as those data becomes available.

Processes that occur after *numt* insertion, such as duplications or deletions of *numts*, can also contribute to *numt* diversity, but there the fate of *numts* just follows that of the genome as a whole. As a perhaps mundane aspect of genomic fate, *numts* and *nupts* are rapidly methylated in higher plants and thus rapidly undergo C-to-T transitions [59]. The same process probably also occurs in animals, but is more difficult to detect because of the paucity of CpG sites in animal mtDNA [70]. *Numts* have no self-replicating mechanism or transposition mechanism; therefore, *numt* duplication is expected to occur in tandem or to involve larger segmental duplication at rates representative for the rest of the genome [23].

In domestic cats, a 7.9-kb mtDNA segment is repeated in 38–76 tandem copies on chromosome D2 [11]. While these repeats were originally suggested as being duplicated pre-insertion, their copy number variability may also result from post-insertion recombination. Additional tandem repeats of 47 bp–long *numt*s appear 18 times on human chromosome 12 [19],[21]. Evidence for *numt* duplications that are not in proximity to other *numts* is present in many genomes [22],[23],[71] and probably happens as part of segmental duplication [23]. However, duplications of recent human-specific *numts* as part of segmental duplication seem to be rare. Four human *numts* showed overlap with segmental duplications. In these cases, *numts* were found in only one of the copies while missing from the others, clearly demonstrating that the *numts* were inserted subsequent to the duplication events [61].

Deletion of *numts* from genomes has not been studied in the same amount of detail as has insertion. However, a recent report in plants shows that *nupts* that are engineered into the genome from transformed plastids are subject to severe instability due to rapid loss [72]. In humans, phylogenetic analyses suggest that the oldest *numt* was inserted 58 million years ago [23]. That suggests that older *numts* have been deleted from the genome, but at the same time, finding similarly ancient *numts* using human mitochondria becomes difficult because of the continuous erosion of phylogentic signal through mutation and the high mutation rate of animal mitochondrial DNA. Similar to recent insertions (Figure 1B) and cases in which the presence–absence pattern of *numts* does not agree with the phylogenetic tree (lineage sorting or reversal) [31], it should be possible to detect recent *numt* losses using a multiple genome alignment when an outgroup is present.

## Correlation between *Numt* Content and Genome Size

Barring a role for differential mtDNA escape into the nucleus as a limiting factor in lineage-specific *numt* frequency (at least in species where multiple copies of mitochondria exist), the finding that *numt* content is strongly correlated with genome size points to the participation of two mechanistically independent processes: integration into the nuclear chromosome and post-insertional processes.

Integration now appears to implicate DSBs. DSBs can arise spontaneously during growth or can be induced by external stimuli such as radiation. Reactive oxygen species (ROS) arising in the mitochondria can also cause nuclear DNA damage [73],[74]. In yeast, it was suggested that increasing the amount of DNA, from diploid to tetraploid, is accompanied by a proportional increase in the fraction of spontaneous DSBs in cells [75]. If this trend is universal (which is a big if), then larger genomes will experience more DSBs. Since *numts* are captured in DSBs, then *numts* would be predicted to appear more often in bigger genomes than in smaller ones (but at a roughly constant per Mb rate). If true, then *numts* should be more common in genomic regions that are prone to DSBs. For example, transcription itself can increase DSBs and genome instability [76]. The enrichments of *numts* in introns versus intergenic regions [30],[42] indicates that an open chromosome is conducive to insertion and thus is consistent with this idea. A further prediction is that *numt* frequency should be higher in regions known to be associated with genome instability as in fragile sites, cells that undergo radiation, and in cancer cells.

Another possible explanation for the correlation between genome size and *numt* content is the previously detected negative correlation between DNA loss and genome size [77],[78]. Larger genomes tend to lose less DNA than smaller ones, as was shown for *Drosophila* and *Laupala*, which vary 11-fold in their DNA content [77]. A negative correlation also exists between genome size and repetitive DNA content [79]. Correspondingly, inaccurate DSB repair after a break-induction in *Arabidopsis* involves large deletions while DSBR of the tobacco genome, which is 20-fold larger, is associated with insertions [80]. Bensasson et al. [17],[43] suggested that *numts* might show similar patterns; animal genomes with more non-coding nuclear DNA would be expected to have more *numts*, while ones with less non-coding DNA will tend to lose them. In other words, this mechanism simply entails a genome-wide tendency to lose DNA in small genomes, such that the *numt* frequency would be independent of DSB frequency, in which case *numt* frequency might be expected to correlate with noncoding DNA amount.

## *Numts* and New Horizons

Over longer evolutionary timeframes, with DNA continuously being transferred from organelles to the nucleus, one might wonder why any DNA has remained in the organelles at all. The reasons for this have to do with the essential bioenergetic function of the organelle [64], namely generating a protonmotive force across the inner mitochondrial membrane with the help of redox chemistry within the inner mitochondiral membrane; the organelle has to have a decisive say in maintaining redox balance throughout the respiratory chain, and this requires retention and regulation of a few genes within the organelle [65]. Indeed, only when organelles fully relinquish their membrane-associated electron transport chains do they fully relinquish their DNA [81].

Over more recent evolutionary timeframes, one finding stands out, namely that about one third (12 out of 40) of those *numts* that were inserted specifically in the human lineage are polymorphic for the presence versus absence of the insertion among human populations (Figure 2). Of course, when the 1,000 genome data for humans becomes available, the number of loci polymorphic for *numts* can be expected to increase.

Future challenges will include gaining a fuller understanding of post-insertion processes at the population genetic level. For example, do *numts* segregate in populations at frequencies that are consistent with neutral, deleterious, or beneficial effects? While there are good reasons to assume neutrality [23], the disease-related phenotypes of several *numts*, as well as the potentially beneficial role that *numts* play in DSBR, indicate that the spectrum of *numt* mutational effects may be broad. More studies on polymorphism for *numts* in human genomes should provide incisive clues. With the sequencing of 1,000 human genomes—and 1,000 *Drosophila*, 1,000 *Arabidopsis*, and many more after that—the data to test many ideas about the evolutionary dynamics of *numt*s are not far away.

A particularly interesting aspect is that *numts* can tell us about the history of the species and which populations or subspecies must have had historically overlapping biogeographic distributions. Neanderthal's *numts* and a scan for Neanderthal mtDNA in a broad sample of human nuclear genome sequences might be an interesting undertaking. An additional fascinating aspect especially in humans, is that polymorphic *numts* potentially provide much more information than just another segregating marker [31], because they can link a given maternally inherited mitochondrial genotype with nuclear DNA polymorphism. The nuclear haplotypes flanking a particular *numt* insertion can tell us which nuclear genotypes and which mitochondrial haplotypes coexisted within the same germline at the particular point in time during which the *numt* was inserted. As such, they offer the opportunity, so far unexplored, to associate nuclear markers with mitochondrial markers back in time and thus to tie mitochondrial with nuclear genome evolution. While recombination within the nuclear genome might put a limit on the detectablility of such associations for *numts* inserted during the early phases of human evolution, this could still potentially represent a rich source of information about human history and admixture to be gleaned from the 1,000 human genome data, and similar endeavours, when it becomes available.
